# Supplementary figures and images for: Persistence of Brucella abortus lineages revealed by genomic characterization and phylodynamic analysis
Source: PLoS Negl Trop Dis. 2020 Apr 14;14(4):e0008235. doi: 10.1371/journal.pntd.0008235 (PMC7182279; doi:10.1371/journal.pntd.0008235)

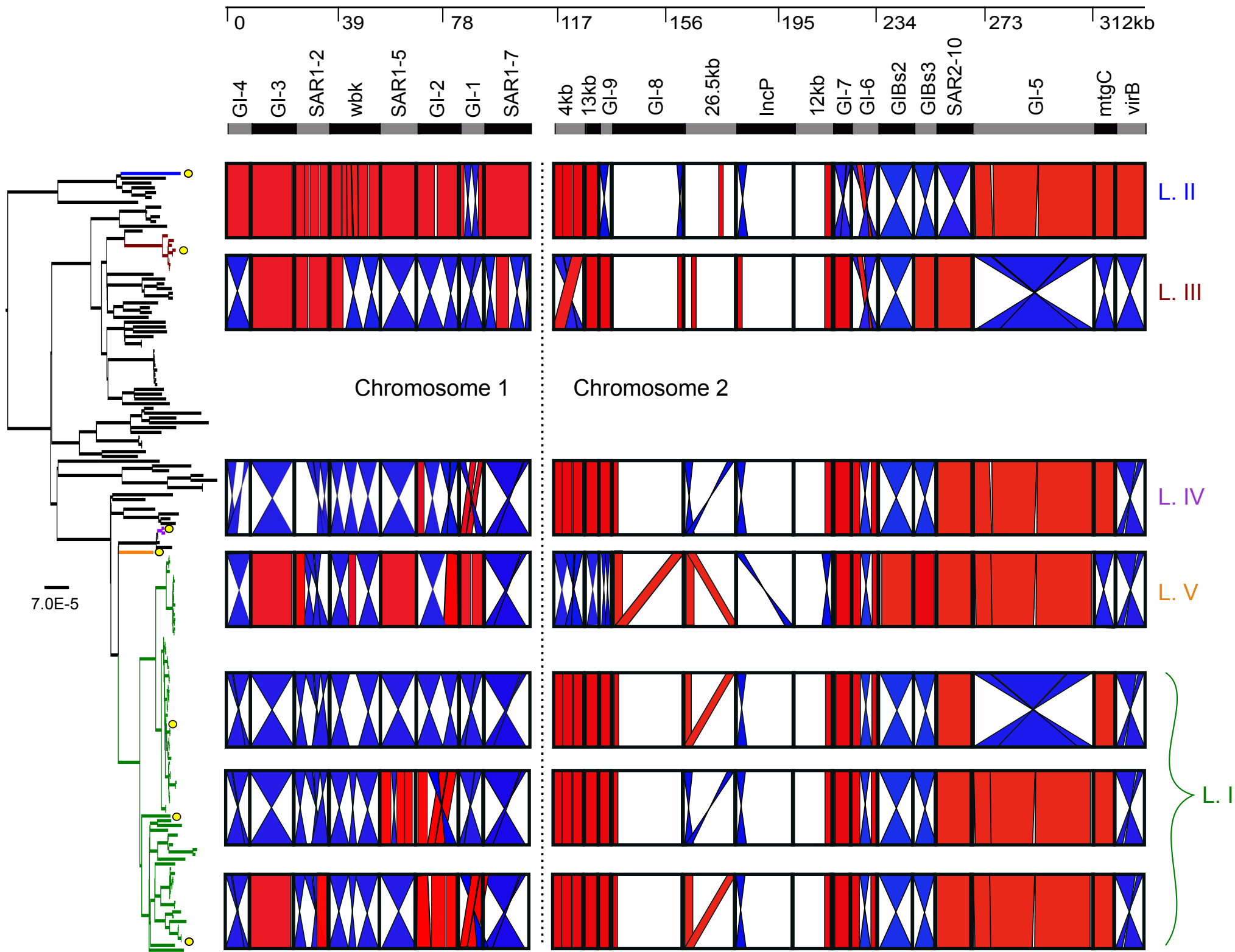

Supplement: S1 Fig — Islands were concatenated and ordered in a pseudo-molecule; this is represented as the upper gray-black blocks. Top coordinates show relative size in bp of each island. Dotted gray line represents the division of both chromosomes. Each comparison box shows the islands’ distribution in the query genomes. Regions present in the same position and order in the genomes (when compared to the pseudomolecule) are shown in red color, and inversions in blue. Absence of segments of the islands are shown as white spaces in the boxes. Independently of the presence of inversions, different rearrangements of the genes included in the islands are observed among the isolates. CR lineages are indicated by the colors of the branches and at the right side of the boxes: lineage I, green; lineage II, blue; lineage III, maroon; lineage IV, purple; lineage V, orange. (PDF) [file pntd.0008235.s004.pdf]

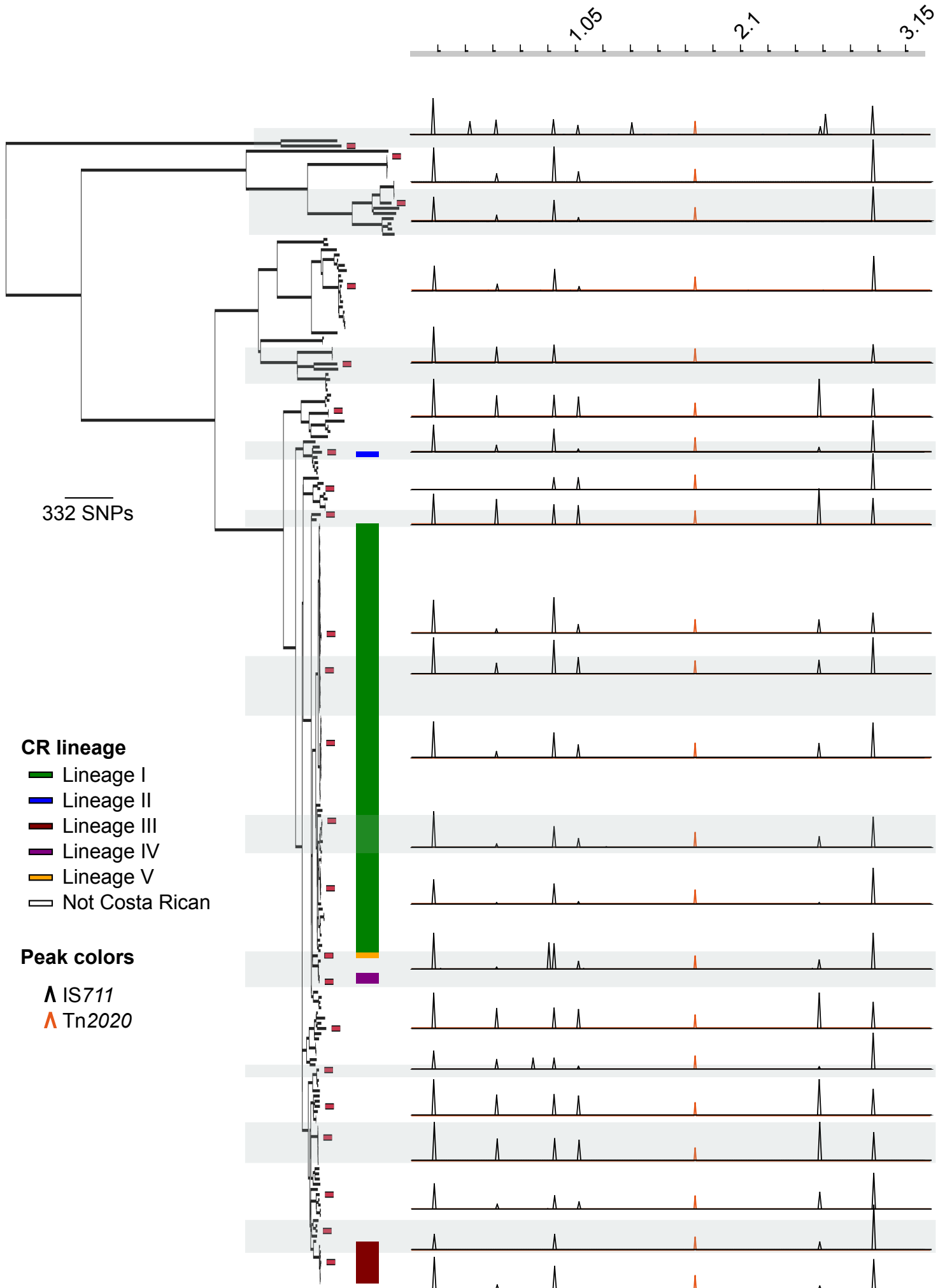

Supplement: S2 Fig — Each peak represents the location of at least 50X coverage, 99% identity IS711 insertion (peak in black color) and Tn2020 (peak in red color). The position in the first and second chromosomes (shown as a concatenated molecule) is indicated by the scale bar (in Mb) above. The smaller maroon bars next to the tips indicate the representative genomes used for the analysis. CR lineages are indicated by a colored bar next to the tips. (PDF) [file pntd.0008235.s005.pdf]
