# Supplementary material for: Persistence of Brucella abortus lineages revealed by genomic characterization and phylodynamic analysis
Source: PLoS Negl Trop Dis. 2020 Apr 14;14(4):e0008235. doi: 10.1371/journal.pntd.0008235 (PMC7182279; doi:10.1371/journal.pntd.0008235)
Supplement: Attachment — Submitted filename: Response to reviewers.pdf [file pntd.0008235.s008.pdf]

PROGRAMA DE INVESTIGACIÓN
EN ENFERMEDADES TROPICALES
Escuela de Medicina Veterinaria, Universidad Nacional,
Apartado Postal 304-3000, Heredia COSTA RICA
Fax: 506/22381298, Tel:506/22380761,
E-mail: catguz@una.cr

Heredia, January the 22th, 2020

Dear Dr. Elaine Maria Seles Dorneles

As requested, enclosed please find files:

1. A word file named “Revised Article with Changes Highlighted” with tracked changes denoting where the text has been changed to answer reviewer comments and minor changes related to text improvement.
2. A word file named “Article file”, which is the clean version submitted the first time and revised by the reviewers.
3. Five tiff files named Fig 1, Fig 2, Fig 3, Fig 4 and Fig 5, which had been previously uploaded to PACE to ensure that figures meet PLOS requirements
4. Four files containing Supplementary info, 3 excel files named: S1_MLVA-16_&WGS_Metadata, S2_Dataset-MLEs, S3_Dataset-SNPsDetails and 1 pdf file named S4_GI_ACT.
5. Due to space constrains, the list of accession numbers is included as supplementary material in excel file S1 and not as a list as suggested.
6. A tiff file name “striking image”, containing a copy of Fig 1 to consider to be featured on the journal website. We think this image is eye-catching and also a good example on the resolution power that the combination of metadata and WGS is providing in the present. This resolution power will help Health Authorities worldwide to better understand the dynamics of pathogens and disease spreading, and therefore to make more precise decisions on how to prevent disease spreading.
7. A word file named “Other file” containing a short caption of the striking image uploaded and credits for this image. The artist has read and agreed to the terms and conditions of the Creative Commons Attribution License at http://journals.plos.org/plosntds/s/content-license

In the following table with 2 columns you will find a detailed list of our responses to the review comments, including the reviewer comment in one column and our response in the next column

Sincerely:

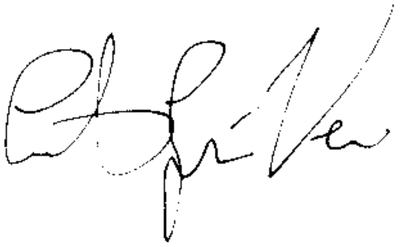

Dra. Caterina Guzmán Verri

Reviewer comment	Response (pages and lines giving according to new version)
Reviewer #3: The objectives of the study clearly articulated with a clear testable hypothesis stated. The description of the isolates was performed in more detail in the results section. Information on host and location of the isolates should be in the methodology section. In the results section, the most important to be presented is not the characterization of the isolates studied as to their basic information, but the outcome of the proposed analyzes.	As suggested by the reviewer, the findings of the analysis are presented in the results section and the description of the isolates including host and location was moved from the results section to the methodology section and referred to S1 and Table 1. This description is **in page 6 lines 151 to 159**, as follows:<br><br>"A total of 188 *B. abortus* clinical isolates recovered in CR during 2003 to 2018 were obtained through the brucellosis national surveillance programs, from Rose Bengal serology test positive animals or humans. Cattle isolates comprised the majority of samples (n= 162), followed by human (n=16) and water buffalo (n=10) (Table 1). Of those, 54 isolates from bovines from the province of Cartago were recovered during an outbreak that occurred from 2003 to 2005 in an 18.7 km^2 and geographically restricted area named San Juan de Chicuá. The isolates were characterized by phenotypic and molecular methods as previously described [8]. WGS was performed for 95 isolates, including all human isolates and the rest selected according to MLVA-16 clustering and geographical distribution (S1 Dataset)."
Reviewer #3: The results are well presented, however, it is recommended to review Figure 3: Please check the color of the subtitle in Clades 1 and 2, it seems to me that Clade 1 should be represented in green, and Clade 2 in blue, following the logic of the previous figures and also due to the amount of isolates in each one.	We thank the reviewer for acknowledging this error. Figure 3 has been corrected consistently with previously used color codes.
Reviewer #2: The conclusions supported by the data presented, but sepecies had no observed in conlusion.	Not sure what the reviewer means. We assumed that is related to host species and not Brucella species, in the same line as the suggestion below from reviewer #3. Accordingly, we have added the following statement in the Discussion section, **page 21-22, lines 620 to 627,**<br>" The presence of two lineages (lineages I and III) where there are genomes from isolates obtained from cattle, buffalo and humans, has not gone unnoticed and seems relevant. Transmission between these species is therefore a possibility in CR. In the case of lineage I, most of the human isolates differ in only 27 SNPs positions, regardless of the origin and time span. To further understand these observations in the context of animal and human infection, a One Health approach is required, with cross-sector collaboration of those involved in such tasks, including public health services, academia, medical and veterinary health agencies, private sector as well as regional and international organizations.”
Reviewer #3: The findings of the	We have modified the discussion section in order to introduce this reviewer

study are in line with the objective and demonstrated the importance and applicability of the results in promoting a better understanding of animal brucellosis epidemiology. Although no results related to human isolates have been discussed, which was acknowledged by the authors, the study makes an interesting contribution to public health. In the context of one health it is essential to recognize the importance of a good understanding of the dynamics of infections in animals in order to prevent disease in human population.	suggestion as follows (Discussion section, **page 21-22, lines 620 to 627):**<br><br>" The presence of two lineages (lineages I and III) where there are genomes from isolates obtained from cattle, buffalo and humans, has not gone unnoticed and seems relevant. Transmission between these species is therefore a possibility in CR. In the case of lineage I, most of the human isolates differ in only 27 SNPs positions, regardless of the origin and time span. To further understand these observations in the context of animal and human infection, a One Health approach is required, with cross-sector collaboration of those involved in such tasks, including public health services, academia, medical and veterinary health agencies, private sector as well as regional and international organizations."
Reviewer #3: Line 118: It is not clear in S1 which 188 isolates were analyzed in this study.	We have reordered S1 so that the costarican isolates are now shown as the first 188 isolates in sheet one. An additional comment referring to additional info related to the isolates or MLVA-16 profiles was added in column "Y".
Reviewer #3: Line 120: Specify how many samples were obtained through the brucellosis national surveillance programs or national hospitals.	The metadata was obtained from the Public and Veterinary Health Authorities. In 10 cases the info is not complete and therefore we cannot be as specific as the reviewer suggested. In Costa Rica, the report of brucellosis cases is mandatory to the corresponding Health Authorities. Since hospitals are part of the Public Health System, and in order to avoid confusion, we have removed "national hospitals" from the sentence **page 6, lines 151 to 153** to read as follows:<br><br>" A total of 188 *B. abortus* clinical isolates recovered in CR during 2003 to 2018 were obtained through the brucellosis national surveillance programs, from Rose Bengal serology test positive animals or humans."
Reviewer #3: Line 149: Explain here the reason for sequencing this isolate with a different technique (Line 273 to 276).	The isolate was sequenced by both Illumina and MinIon. To clarify this, we have modified the sentence from " To avoid the loss of any information contained at contig breaks from human isolates, we confirmed our findings by sequencing a representative human isolate (babohCR175) using minION Oxford Nanopore (S1 Dataset)" to:<br><br>" To avoid the loss of any information contained at contig breaks, and/or differences at indel level from human isolates, we confirmed our findings by also sequencing a representative human isolate (babohCR175) using minION Oxford Nanopore (S1 Dataset)." **(page 13, lines 373 to 376)**<br><br>Additionally and for clarification purposes, the following sentences (page 13, lanes 271 to 273 in previous version) " Interestingly, of those 83 isolates, nine obtained from humans clustered together and were separated by 27 SNPs from the other genomes included in lineage I (S3 Dataset). We did not detect a connection between these alternative alleles in each SNP and metabolic pathways" are now **(page 13, lines 370 to 373):**<br><br>"Interestingly, of those 83 isolates, fourteen obtained from humans were found within this lineage. Of those, nine coming from different country regions are closely related and separated by only 27 SNPs positions from the other genomes included in lineage I (S3 Dataset). We did not detect a connection between these alternative alleles in each SNP position and metabolic pathways."
Reviewer #3: Line 154: In supplementary material S1,	The list of genomes used for the phylogenetic study is in supplementary material S1, sheet named "WGS metadata". When filtered by "This study" within the

when using the "This study" filter only 53 records are found. Incompatible or unintuitive to find which are the 95 genomes used in this analysis. Please check and correct.	costarican isolates, 95 genomes are shown. When filtered by "This study" in sheet named "MLVA profiles & metadata", where the isolates used for MLVA-16 analysis are shown, the number of hits is 53, which is probably what the reviewer found.<br>We have reordered S1 so that the costarican isolates appear first. We have also corrected the number of total *B abortus* genomes used for the phylogenetic reconstruction, from 221 to 219 and clarified the number of other *Brucella* genomes used, in the Methods section as it follows **(page 8, lines 215-220):**<br>"A total of 219 *B. abortus* genomes were used in this analysis; 101 of those generated during this study. The detailed information and metadata of the genomes is presented in S1 Dataset and includes 95 CR isolates: 76 from cattle, 3 from water buffalo, and 16 from humans. These were analyzed alongside 8 genomes from reference strains from other *Brucella* species (*B. canis*, *B. ceti*, *B. melitensis*, *B. microti*, *B. neotomae*, *B. ovis*, *B. pinnipedialis* and *B. suis*) and two *Ochrobactrum* species were used as an outgroup."<br><br>And accordingly, in the results section **(page 11-12, lines 304-306):**<br><br>"A SNP matrix, including 322,266 sites from 219 *B. abortus* genomes, 8 from other *Brucella* species and two *Ochrobactrum* genomes as an outgroup, was generated and used to produce a maximum likelihood phylogenetic tree, with *B. abortus* 9-941 as a reference."
Reviewer #3: Line 177: Quote abbreviation for CoDing Sequence (CDS) in the first time it appears in text.	The abbreviation has been added as requested**, line 243, page 8**
Reviewer #3: Line 278: As a suggestion, it would be interesting for the circles to have a size proportional to the number of isolates they represent.	We agree with the reviewer's recommendation. Figure 2 and its legend **(lines 378-385, page 13)** have been updated accordingly:<br><br>"**Fig 2.** Map of CR indicating the regions from which *B. abortus* was isolated (circles). Circle sizes are proportional to the number of isolates recovered from each geographical point, and the colors correspond to lineages I-V. The herd prevalence determined by Rose Bengal test for bovine brucellosis in each socioeconomical region is shown in parenthesis [8]. Prevalence in water buffalo is estimated in 21.7% [9]. Lineage I and III are dispersed along the territory, but are mainly present in the North-East region of CR, where livestock expansion predominates. Map modified from Wikimedia Commons (commons.wikimedia.org), Creative Commons CC0 License."
Reviewer #3: Line 279: Lineage II is represented with a blue sign only in the province of Chorotega. Lineage III in turn seems to be more widely distributed. Please check.	The typo has been corrected **in line 382**, as indeed it referred to lineage III, not II as follows (page 13, lines 382-383)<br><br>Lineage I and III are dispersed along the territory, but are mainly present in the North-East region of CR, where livestock expansion predominates.
Reviewer #3: Line 297 and 298: Phrase more appropriate in discussion section than in presentation of results.	Phrase was moved and put in context, as suggested to discussion section, **pages 18-19, lines 555-559** as follows:<br>"We used genomic characterization and phylodynamic analysis of *B. abortus* isolates as a model to understand the evolution of brucellosis in CR, in order to detect the origins and spread of circulating strains. MLVA has been used widely for genotyping studies worldwide [53,54]. In this study, the MLVA-16 results obtained did not match those obtained by phylogeny based on WGS. This difference, could be explained by the higher resolution power provided by WGS and homoplasy of *B. abortus* VNTR markers, as previously proposed [55–58]."
Reviewer #3: Paragraphs of lines 309 to 316: Avoid excessive	The use of "we" has been reduced as suggested.

use of the pronoun "we".	
Reviewer #3: Line 331 to 334: Phrase more appropriate in discussion section than in presentation of results. Attention, please insert reference to Kamath et al., 2016.	Is it possible that the reviewer meant lines 428 to 432? We inferred that this could be the case since this reference is missing in this part of the manuscript and present in line 331. In any case, as suggested by the reviewer, the following phrase was removed from the results sections:<br><br>"slower than the 1.4 $\times 10^{-7}$ (95% HPD interval:1.09 $\times$ 10^{-7}–1.73 $\times$ 10^{-7}) substitutions per site per year previously reported [49]."<br><br>This phrase is now in the discussion section, with the proper cite and within the following context: **Discussion section, page 20, lanes 592 to 596**:<br><br>"Here we estimated a lower substitution rate for *B. abortus* than Kamath et al. 2016 (61). They reported 1.4 $\times$ 10^{-7} substitutions per site per year (95% HPD interval:1.09 $\times$ 10^{-7}–1.73 $\times$ 10^{-7}); interestingly the 95% HPD of our estimated rate overlaps with their median value. Both results are similar to the 10^{-7} to 10^{-8} mutations per site per year of the intracellular microorganism *Mycobacterium tuberculosis* (62–64)."
Reviewer #3: Line 373 to 376: Phrase more appropriate in discussion section than in presentation of results.	We thank the reviewer for the suggestion. We have decided to keep such phrase in the results section since it is describing the results presented in Figure 5. This figure is an original result, gathering information from different sources and provides an appropriate context for the time-structured phylogenetic analysis.
Reviewer #3: Although there is no clear explanation for the results observed for human isolates, it is important that the authors at least hypothesize what was observed. The results should at least provide guidance and suggest possible strategies for further investigations in the epidemiology of human brucellosis.	We agree with the reviewer and in fact, we are also intrigued by this result. After several discussions with epidemiologists and bacterial genomics experts, one possible interpretation of such result is the emergence of a new cluster of *B. abortus* that is infecting specifically human hosts. Sexual transmission might be a route of infection. Due to the close contact of humans to infected domestic bovines in Costa Rica, this hypothesis is very difficult to test and therefore we decided to leave it out of the manuscript, in order not to deviate attention from the main message.<br>As for the possible strategies, they were included, described above and repeated here:<br><br>(Discussion section, **page 21-22, lines 620 to 627):**<br><br>" The presence of two lineages (lineages I and III) where there are genomes from isolates obtained from cattle, buffalo and humans, has not gone unnoticed and seems relevant. Transmission between these species is therefore a possibility in CR. In the case of lineage I, most of the human isolates differ in only 27 SNPs positions, regardless of the origin and time span. To further understand these observations in the context of animal and human infection, a One Health approach is required, with cross-sector collaboration of those involved in such tasks, including public health services, academia, medical and veterinary health agencies, private sector as well as regional and international organizations."
1. Lines 251-252: "The phylogenetic reconstruction showed clustering patterns consistent with the country of origin of the isolates (Fig. 1)". The data presented in Fig. 1 do not support this statement. Also, except for the Costa Rica isolates, the country of origin of other isolates is not shown.	We agree with the reviewer. The statement is not supported by Fig. 1 but rather by the data presented in the microreact version, available at https://microreact.org/project/BJJ4H3H-E. Fig. 1 is a zoom of what is represented in the microreact project, showing mainly the Costa Rica lineages. In this figure, to facilitate context visualization, a simpler color code was chosen with only continents represented, not countries. Accordingly, text has been modified as follows (**page 12, lines 342 to 345):**<br><br>"The phylogenetic reconstruction showed clustering patterns consistent with the country of origin of the isolates (https://microreact.org/project/BJJ4H3H-E). When looking at the isolates found in Costa Rica (Fig 1) and according to hierBASP (S2 Dataset) and BEAST, there are at least five distinct lineages currently circulating in the country."

2. Fig. 1: Strain RB51 is a derivative of strain 2308. It is surprising to see these two strains separated into two different clusters in the phylogenetic tree. Reevaluation of the detected SNPs between these two strains is recommended. Did the authors sequence strain RB51 isolated from the abortion cases?	In spite of being located at different clusters, the distance between RB51 and 2308 represented in Fig 1 is only 21 SNPs (see scale info in Fig 1). No, the RB51 genome used in the phylogenetic reconstruction was taken from public databases, accession number GCA_000366025.1 (see info in S1).
3. Fig. 3: Figure legend: Clade I and II colors are switched.	We thank the reviewer for this observation. Fig 3 has been updated and corrected accordingly.
4. Table 2: What do these numbers represent? Specific years CE/AD? If so, why include the<br>decimal values?	The numbers represent AC years. The decimals were removed from the table and table title was modified as follows **(page 16, lines 479-480):**<br><br>**"Table 2.** Highest posterior distribution interval and time (shown in AC years) to the most common recent ancestor (MRCA) from the five Costa Rican lineages."
5. Line 374: "900s" should be "1900s".	Indeed, the typo has been corrected in **page 18, lane 534.**
6. Line 374: References 50 and 51 do not appear to be relevant to this statement.	We thank the reviewer for noticing this error. The references were renumbered
7. Lines 379-381: Reference 53 is not pertinent.	We thank the reviewer for noticing this error. The references were renumbered.
8. Line 389: Reference 54 is not the correct reference for this statement.	We thank the reviewer for noticing this error. The references were renumbered.
9. All references in Discussion section must be cross checked for their relevance to the stated information.	We thank the reviewer for noticing this error. The references were renumbered.
1. orders of line 83-86 and line 87-90 need exchange, for example: CR is a tropical……, The only prevalent Brucella species in 84 domestic livestock of CR.	The paragraphs have been reordered as suggested in **page 4, lines 88-95** as follows:<br><br>"Costa Rica is a tropical and subtropical Central American country with a land area of ~51,100 km^2. Socioeconomically, the country is divided in six regions: Northern, Central, Brunca, Chorotega, Caribbean Huetar and Central Pacific [7]; the Northern and Central regions are the main livestock farming areas. The only prevalent *Brucella* species in domestic livestock of Costa Rica (CR) is *Brucella abortus*, with a seroprevalence in cattle close to 11% and to 21.7% in water buffalo farms [8,9]. No information of brucellosis in CR terrestrial wildlife is available. Brucellosis prevalence in cattle has been reported as 0.5–10% in Latin American countries, and from 4–11%

	in Central American countries [10–12]”
2. line 97-98 “an increasing number of human cases have been reported” need further discussion.	This paragraph has been modified as follows page 5, lines 121 to 125:<br><br>"Brucellosis became a notifiable disease in CR in 1915, after the first isolation of “Bang´s bacillus” from the blood of a human patient [15,16]. More recently, and thanks to improved surveillance and diagnosis of the disease by public health authorities [8].an increasing number of human cases have been reported, with positive hemocultures and description of the first human cases caused by *Brucella neotomae* [17,18]".
3. The species/biovar of 188 strains had no identified? This context is important to understand the epidemiology of abortus brucellosis, please supplement.	In Costa Rica, and according to the OIE guidelines: "Species and biovar identification requires elaborate tests (such as phage lysis and agglutination with anti-A, -M or -R monospecific sera), the performance of which should be left to reference laboratories with accredited expertise in these methods" (https://www.oie.int/fileadmin/Home/eng/Health_standards/tahm/3.01.04_BRUCELLOSIS.pdf, accessed Janruary the 21th)<br>Due to lack of technical expertise (most technicians who were training in these techniques are retiring) and lack of budget, biovar identification was not performed. A middle term goal in our research is to help in the transition from conventional typing techniques to genomics based techniques. In the near future, when funds are available, we are planning to perform biotyping of these isolates and compare them with the phylogenetic analysis shown in this manuscript.
3. How to selection 95 strains for WGS, random? or what is reference for this choose?	A sentence indicating such information has been added in the methods section as follows **(page 5, lines 157 to 159):**<br><br>"WGS was performed for 95 isolates, including all human isolates and the rest selected according to MLVA-16 clustering and geographical distribution (S1 Dataset)."
4. we had on found results on MLVA analysis?	We apologize since we are not sure what the reviewer meant with this question
5. relationship relative between biovar and lineage?	As mentioned before, a middle term goal in our research is to help in the transition from conventional typing techniques to genomics based techniques. In the near future, when funds are available, we are planning to perform biotyping of these isolates and compare them with the phylogenetic analysis shown in this manuscript.
6. more discussion and improve are need in “54 isolates, recovered during an outbreak during 2003-2005”	Unfortunately, all the info we have related to such outbreak is described in S1. This info is very brief and therefore we are not able to discuss more about it as requested by the reviewer.
7.suggest that species/biovar of strains should be list in Fig. 2 each area, serology positive rate of abortus brucellosis?	Fig 2 was modified in order to include the seroprevalence shown by Rose Bengal test in previous studies (Hernández-Mora et al. PLoS One. 2017;12(8):1–17 and Hernández-Mora et al. PLoS One. 2017;12(8):e0182644). The legend has also been modified as indicated in **page 13, lines 378-385:**<br><br>“Fig 2. Map of CR indicating the regions from which *B. abortus* was isolated (circles). Circle sizes are proportional to the number of isolates recovered from each geographical point, and the colors correspond to lineages I-V. The herd prevalence determined by Rose Bengal test for bovine brucellosis in each socioeconomical region is shown in parenthesis [8]. Prevalence in water buffalo is estimated in 21.7% [9]. Lineage I and III are dispersed along the territory, but are mainly present in the North-East region of CR, where livestock expansion predominates. Map modified from Wikimedia Commons (commons.wikimedia.org), Creative Commons CC0 License.”<br>As stated above, biotyping info is not available at this moment.
8. discussion need improved, for	As suggested by reviewer #3, we have modified the discussion section to

example :10 human isolated had no discussion,<br>water buffalo is livestock or wildlife in CR, what role in transmission of abortus.	comment the results related to the human isolates, possible scenarios of disease spread within species and prevention strategies as follows:<br><br>(Discussion section, **page 21-22, lines 620 to 627):**<br><br>" The presence of two lineages (lineages I and III) where there are genomes from isolates obtained from cattle, buffalo and humans, has not gone unnoticed and seems relevant. Transmission between these species is therefore a possibility in CR. In the case of lineage I, most of the human isolates differ in only 27 SNPs positions, regardless of the origin and time span. To further understand these observations in the context of animal and human infection, a One Health approach is required, with cross-sector collaboration of those involved in such tasks, including public health services, academia, medical and veterinary health agencies, private sector as well as regional and international organizations."<br><br>Regarding water buffalo status in Costa Rica, we have added the following information in the introduction section **(page 5, lines 117-120):**<br><br>"On the other hand, records indicate that the introduction of water buffalo occurred in 1974 [14]. The first *B. abortus* isolates recovered in Costa Rica from buffalo are from 2018, reported in this study.'
