# Supplementary material for: Persistence of Brucella abortus lineages revealed by genomic characterization and phylodynamic analysis
Source: PLoS Negl Trop Dis. 2020 Apr 14;14(4):e0008235. doi: 10.1371/journal.pntd.0008235 (PMC7182279; doi:10.1371/journal.pntd.0008235)
Supplement: Attachment — Submitted filename: PLoSNTD-2019.docx [file pntd.0008235.s006.docx]

In this manuscript, the authors report the genomic and phylogenetic analyses of selected *Brucella abortus* isolates from Costa Rica, and propose a potential timeline and external sources of *B. abortus* introduction into the country. The manuscript is well-written and the interpretation of the results is appropriate.

Comments:

1. Lines 251-252: “The phylogenetic reconstruction showed clustering patterns consistent with the

country of origin of the isolates (Fig. 1)”. The data presented in Fig. 1 do not support this statement. Also, except for the Costa Rica isolates, the country of origin of other isolates is not shown.

2. Fig. 1: Strain RB51 is a derivative of strain 2308. It is surprising to see these two strains separated into two different clusters in the phylogenetic tree. Reevaluation of the detected SNPs between these two strains is recommended. Did the authors sequence strain RB51 isolated from the abortion cases?

3. Fig. 3: Figure legend: Clade I and II colors are switched.

4. Table 2: What do these numbers represent? Specific years CE/AD? If so, why include the decimal values?

5. Line 374: “900s” should be “1900s”.

6. Line 374: References 50 and 51 do not appear to be relevant to this statement.

7. Lines 379-381: Reference 53 is not pertinent.

8. Line 389: Reference 54 is not the correct reference for this statement.

9. All references in Discussion section must be cross checked for their relevance to the stated information.

1. Characterization of the rough mutant is incomplete. Additional experimental details such as primer sequences used to create the deletion construct and for verification of the gene deletion must be provided. In addition to the rough phenotypic characterization, the absence of O antigen synthesis in the mutant strain must be demonstrated by Western blot analysis or LPS analysis. Complementation studies could be done to confirm that the rough phenotype was because of the *wboA* gene deletion. The absence of anti-smooth LPS antibodies in mice vaccinated with the rought strain needs to be confirmed.

2. Dendritic cell experiments: If the intent is to examine the differences in innate immune activation by the vaccine candidates, why only measure TNF-alpha production? What about other important cytokines such as IL-12? Fir

3. If the goal is to develop a vaccine that could provide protection against all pathogenic *Brucella* species, why not include *B. abortus* and *B. melitensis* challenges in the study?

Other comments:

1. Dendritic cell preparation and infection experiment (lines 130-134): The gentamicin treatment was carried out at what time point? Prior to collecting culture supernatants?
2. Protection in mice (line 149): what was the route of VTRS1 vaccination? Winter et al used 5 x 10^4^ CFU vaccine dose via intravenous route, not intraperitoneal route.
3. Including Figure 1 in the main article requires justification. If this figure needs to be included in the main article, then it should also include photos of crystal violet staining and monoclonal antibody agglutination test results.
4. Innate immune response (lines 201-208): What were the intracellular CFUs at 4 h, 24 h and 48 h post infection? Why not show the TNF-alpha production data at 24 h and 48 h post infection?
5. Figure 3. The data points at 0 day should be removed from the figure. You did not determine the spleen bacterial load on day 0. The data can be shown in bar graphs instead of line graphs.
6. Protection against challenge (lines 227-234): State the log protection units in the text.
7. Antibody titers (Figure 6): It appears that the IgG2a levels are too low in the figure. (Were the assays optimized properly?) The statement “The subtyping shows the significant role of IgG2a subisotype and not that of IgG1” is not supported by the data.
8. Lines 268-269: no genotypic confirmation was provided in the manuscript.
9. In the experiment with different vaccine doses, it is incorrect to conclude that 10^2^ and 10^4^ doses did not induce a protective immune response. It is relevant to note the timing of the challenge relative to the clearance of the vaccine organism. Challenge occurred at least 4 weeks after the clearance of the vaccine organism in case of 10^2^ and 10^4^ doses, after 2 weeks in case of 10^6^ dose and on the same day in case of 10^8^ dose.
