# Supplementary material for: Persistence of Brucella abortus lineages revealed by genomic characterization and phylodynamic analysis
Source: PLoS Negl Trop Dis. 2020 Apr 14;14(4):e0008235. doi: 10.1371/journal.pntd.0008235 (PMC7182279; doi:10.1371/journal.pntd.0008235)
Supplement: Attachment — Submitted filename: PNTD-D-19-01458 comments.docx [file pntd.0008235.s007.docx]

This article summarized 188 B. abortus isolates from CR recovered from three host, from 2003 to 2018, then whole genome sequencing (WGS) was performed in 95 of them. They founding that introduced in 100 years ago and are widely spread throughout the country, in contrast to new introductions that seemed to be more geographically restricted. This study model can provide a comprehensive insight for control and research of many middle- and low-income countries. The manuscript needs more improvement as follows:

1. orders of line 83-86 and line 87-90 need exchange, for example: CR is a tropical……，The only prevalent Brucella species in 84 domestic livestock of CR.

2. line 97-98 “an increasing number of human cases have been reported” need further discussion.

3. The species/biovar of 188 strains had no identified? This context is important to understand the epidemiology of abortus brucellosis, please supplement.

3. How to selection 95 strains for WGS, random ? or what is reference for this choose?

4. we had on found results on MLVA analysis?

5. relationship relative between biovar and lineage?

6. more discussion and improve are need in “54 isolates, recovered during an outbreak during 2003-2005”

7.suggest that species/biovar of strains should be list in Fig. 2 each area, serology positive rate of abortus brucellosis?

8. discussion need improved, for example :10 human isolated had no discussion,

water buffalo is livestock or wildlife in CR, what role in transmission of abortus.
