# Supplementary material for: Persistence of Brucella abortus lineages revealed by genomic characterization and phylodynamic analysis
Source: PLoS Negl Trop Dis. 2020 Apr 14;14(4):e0008235. doi: 10.1371/journal.pntd.0008235 (PMC7182279; doi:10.1371/journal.pntd.0008235)
Supplement: Attachment — Submitted filename: Comments.docx [file pntd.0008235.s009.docx]

Comments:

The manuscript has been improved.

Minor reversion before publication:

1 Abstract: “Our results show circulation of five B. abortus lineages in CR” suggest: Our results show circulation of five B. abortus lineages (Ⅰ- Ⅵ) in CR,

2 The dominant CR lineage was probably introduced at the end of the 19th century suggest: The dominant CR lineage (Ⅰ or others). Similarly, “One lineage……”, “Other lineages……”，

“same lineage”, please definite and clarify.

3 line 117 Brucella isolates: the authors should better describe the bacteriological methods for isolation, identification and characterization of Brucella species and biovars.

4 line 130, “MLVA and whole genome sequencing”: suggest respective describe these methods, especially, it should be included information about the MLVA markers used in this work and the respective information about allele assigning. Results, data interpretation and discussion are, in general, adequate.

5 results line 300-305, suggest :MLVA analysis results will be as independent section, please don't mix in the genome analysis section.

6 Table: all table is not three-line table, please check journal’s author guidance.

7 Some limitations listed of this research are needed. such as: “Our results show circulation of five B. abortus lineages in CR, phylogenetically related to isolates from the United States, United Kingdom, and South America”. Suggest need for data on animal movements, for example: how many animals were introduced to CR………. Moreover, strains from each region were imbalance and investigation information in serology is lack.
